# Experimental Study of Soft Ballistic Packages with Embroidered Structures Fabricated by Using the Tailored Fiber Placement Technique

**DOI:** 10.3390/ma15124208

**Published:** 2022-06-14

**Authors:** Maciej Gloger, Zbigniew Stempien

**Affiliations:** Institute of Textile Architecture, Lodz University of Technology, Żeromskiego 116, 90-924 Łódź, Poland; zbigniew.stempien@p.lodz.pl

**Keywords:** ballistic packet, para-aramid yarn, embroidered fabric, tailored fiber placement method

## Abstract

Textile ballistic shields are the basis of protection against bullets and fragments with low kinetic energy. They are usually made of para-aramid fabrics or unidirectional structure (UD) sheets of ultra-high molecular weight polyethylene (UHMWPE). The aim of the research presented in the article was to obtain ballistic packages made of embroidered structures and to compare their ballistic properties with those of woven structures in terms of deformation of the standardized ballistic substrate after impact with a 9 mm bullet at a velocity of 380 ± 3 m/s. Using the tailored fiber placement method, embroidered structures were fabricated by embroidering two sets of para-aramid threads at an angle of 90°. As the woven structures, the use of para-aramid fabric made of the same yarn and with a surface weight comparable to that of embroidered structures was adopted. Ballistic packages consisted of 26 layers in five variants, also taking into account the hybrid arrangement of woven and embroidered layers. Ballistic tests have shown that the best ballistic properties have hybrid packages made by folding 13 woven and then 13 embroidered layers, where the maximum deformation of the plasticine substrate is below 23 mm. The conducted research confirmed that embroidered structures in appropriate combination with woven structures can significantly improve the ballistic properties of textile packages.

## 1. Introduction

Textile ballistic shields are the basis of human protection against bullets fired from handguns or fragments with low energy of destruction. Their effectiveness is related to preventing the overshoot of the ballistic package and limiting the ballistic trauma resulting from the quick-changing lateral deformation of the package in the area of the bullet impact. They are mainly made of para-aramid fibers and UHMWPE. It should be noted that in addition to the unique properties of these materials, there are also various mechanisms that must be considered in order to increase the ballistic performance of the final ballistic package. A simple method to improve ballistic protection against ballistic impact is to add more layers in different settings during the production of ballistic panels [1,2]. Although increasing the number of layers of a ballistic shield improves its ballistic performance, it also affects the overall weight and flexibility of the target shield [2]. At present, aramid fabrics with a plain weave or non-woven UD based on polyethylene fibers are used as layers of ballistic packages.

The 2D plain weave fabric is the simplest and most widely used fabric in ballistic applications. It is made by weaving two types of yarns along the warp and weft. Two-dimensional fabrics can be layered to design structures of different thicknesses. Multilayer structures can be sewn together, for example, only to the inside from the edges; to the center from the edges and in the shape of a diamond; inward from the edges, and then on a bias in one or more directions. The use of stitching increases the ballistic resistance and puncture resistance, especially by reducing the deformation of the rear surface, but it also has the disadvantage of increasing the stiffness of the panel, which makes it difficult to use the panels, especially in protective vests [3,4]. Unidirectional laminates are another 2D fabric structure that finds ballistic applications. These structures consist of unidirectional sandwich sheets that are mutually rotated and joined together. The sheets are made by pre-impregnating straight and parallel fibers with resin or rubber. Fibers in unidirectional laminates have different orientation in different layers and the same orientation in one layer [5,6]. Multiaxial fabrics are also used in soft ballistic shields. Compared to traditional biaxial fabrics, multiaxial fabrics may contain more than two interwoven thread patterns in their structure. When the bullet hits the fabric, the multiaxial shock wave propagates radially, as a result of which the fabric area absorbs more of the kinetic energy of the bullet than the biaxial fabric. Such a distributed kinetic energy of the bullet may have a significant impact on the parameters of the resulting deformation cone, as well as on the physiological effects of a ballistic shock. Tests evaluating the ballistic effectiveness of packages consisting of a biaxial and a triaxial fabric were carried out by determining the minimum number of layers ensuring that the package would not be shot through. It was found that the minimum number of layers having protective properties in a package of biaxial fabrics was lower than that of a package of triaxial fabrics. It was the result of the interaction of hexagonal holes in the structure of the triaxial fabric, which makes it very transparent [7,8]. In addition to 2D fabrics, 3D orthogonal fabrics, 3D fabrics with full/partial interlacing, and multi-axis 3D fabrics are also used in soft ballistic covers [9,10]. Three-dimensional fabrics (mainly 3D with warp blocking) show high efficiency in ballistic protection with high flexibility and low weight [11] compared to 2D woven structures [12]. However, the problem of 3D fabrics may be their production, due to their complex structure [13,14].

One technique for forming flat textile structures not yet considered for ballistic shield applications is embroidery. This technique is traditionally known as the conventional technique of decorative textile processing, which is found in almost every culture in the world and has its origins in ancient civilizations. As part of the industrial revolution, embroidery entered mass production, and the scope of its technical applications has significantly expanded with the technological progress [15]. One of the embroidery technologies in this field is tailored fiber placement (TFP). This method provides the possibility of two-dimensional yarn laying on the substrate in a programmed orientation, the creation of three-dimensional structures [16,17] and gives great possibilities in designing the structure and shape of the product. Depending on the technical specification of the embroidery machine and the technical requirements of the project, many different materials can be laid and embroidered on the selected substrate, such as carbon fiber [18] and flax rovings [19], electrically conductive metal yarns [20,21,22,23], wires [24], coated man-made fiber-based yarns [25,26,27], etc. Due to these possibilities, the embroidery technique is increasingly used in composite engineering, medical textiles and e-textiles. In engineering, the composite with embroidery allows for obtaining structures that are characterized by anisotropic properties due to the significant influence of fiber orientation on stresses in structural elements [17]. Generally, composites with programmable strength properties can be used where it is not necessary to transfer loads on the entire surface of the composite, but only on selected fragments, such as beams, ribs, and mesh stiffeners [19]. The composites obtained in this way can be used in the automotive industry, for example, as a car spare wheel, having a lower weight than its steel counterpart, alternator covers, and elements reinforcing the seat belt attachment points [28]. On the other hand, in the aviation industry, composites based on embroidered structures have been used as a conformal structure of a carrier antenna (CLAS), providing, on the one hand, Wi-Fi communication and, on the other hand, radar scanning of space in order to avoid collisions [29,30,31,32,33]. Embroidery is also an enormous asset in the development of consumer and flexible electronics in the field of e-textiles. The use of electroconductive materials in embroidery enables the interconnection of textiles with electronics. The embroidery technique allows the deposition of LEDs [34] on textile substrates, electrically conductive yarns to create electrically conductive lines [35], embroidered antennas and radio frequency identification (RFID) tags [21,36], embroidered temperature sensors [37], embroidered humidity sensors [20,23], and pressure sensors [38,39]. There are also known attempts to fabricate magnetic coils [17,40,41], embroidered current collectors for lithium-iron-phosphorus batteries [42] or embroidered fabric electrodes in supercapacitors [27]. In the production of medical textiles or tissue engineering, the use of embroidery is a new technology successfully used to develop hernia meshes [19], stents for the repair of abdominal aortic aneurysms [43], intervertebral disc implant [43], glucose detection sensors [25,44], sweat lactate [44], gas-based electrochemical sensors for rapid quantification of wound biomarkers [45], ECG [26] and EMG monitoring [22], and bioelectrical impedance analysis [46].

It seems that the advantage of free formation of an embroidered structure by means of TFP technology can also be used to obtain ballistic packages with even better properties than those noted for packages made of woven and UD structures. The aim of the research presented in the article was to obtain ballistic packages made of embroidered structures and to compare their ballistic properties with woven structures in terms of deformation of the standardized ballistic substrate and the mechanisms of layer destruction after impact with a 9 mm Parabellum bullet at a speed of 380 m/s.

## 2. Materials and Methods

**Figure 1:**
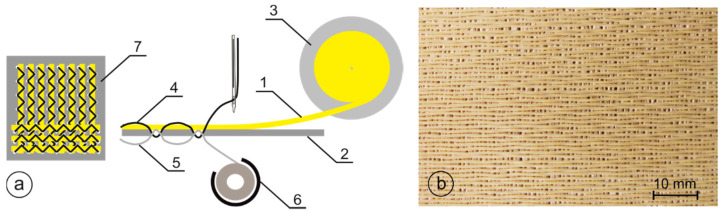
Production of embroidered structures: (**a**) a diagram of the embroidery process (1—para-aramid yarn, 2—PP spun-bonded nonwoven substrate, 3—para-aramid yarn bobbin, 4—main thread zigzagged, 5—thread bottom, 6—swivel hook, and 7—embroidered structure), (**b**) view of the embroidered structure produced.

The commercially available Twaron CT709 Microfilament 930 tex f1000 woven fabric (Teijin Aramid, Arnhem, The Netherlands) was used for the tests, with the number of threads in both systems of 105 threads/dm and the surface weight of 200 g/m^2^. The embroidered structures of size of 20 cm × 20 cm were made of the yarn Twaron Microfilament 930 tex f1000, the same yarn from which the selected woven fabric Twaron CT709 was made, by using the JCZA 0109-550 embroidery machine (ZSK Stickmaschinen, Krefeld, Germany) equipped with the tailored fiber placement head, according to the diagram shown in Figure 1a. During fabrication, para-aramid yarn (1) was fed from the bobbin (3) and attached with the main thread (4) and bottom thread (5) to the PP spun-bonded non- woven fabric (TEXFIL, Łódź, Poland) (2) with a zigzag stitch. The use of a non-woven fabric is necessary due to the specificity of forming an embossed structure, where a substrate is required to which the threads are attached. The areal weight of the nonwoven substrate used was 80 g/m^2^. As main and bottom threads, the thread TYTAN 360 (Ariadna, Łódź, Poland) was used. Its average linear weight and strength at break was equal to 85 dtex and 480 cN, respectively. By using the tailored fiber placement technique, two sets of para-aramid yarns with the number of threads in these both sets of 106 threads/dm were embroidered at an angle of 90° between them (7). The number of threads was iteratively selected so as to achieve the surface weight of the Twaron component in the embroidery fabric similar to the surface weight of the woven fabric Twaron CT709. For the final value of the number of threads equal to 106 threads/dm, the mass of the Twaron component in the embroidery fabrics was 203 ± 5 g/m^2^.

The transverse and longitudinal stroke of the needle when embroidering with a zigzag stitch were 1 and 4 mm, respectively. An exemplary view of the produced embroidered structure is shown in Figure 1b.

**Figure 2:**
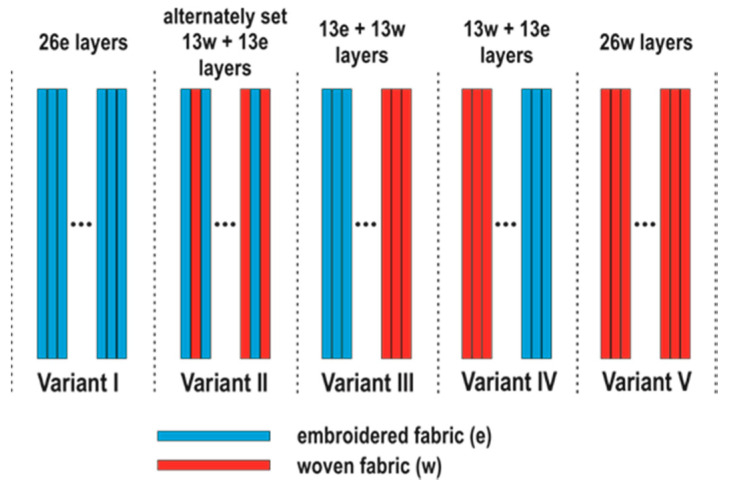
Variants of execution of ballistic packages.

The woven and embroidered structures were used to make five variants of ballistic packages consisting of 26 layers with a size of 20 cm × 20 cm. Such a size of the packages was adopted by taking into account the assumed testing procedures and production time of the embroidered layers. The variants of packages were arranged as follows: Variant I—26 embroidered layers, Variant II—13 embroidered and 13 alternating woven layers, Variant III—13 embroidered layers on the front and 13 woven layers on the back, Variant IV—13 woven layers on the front and 13 layers embroidered on the back, Variant V—26 layers woven (Figure 2). Variant I and Variant V were chosen to compare packages composed only of fabric and embroidery. Variants III and IV were chosen, expecting a high perforation rate for a package made of embroidered structures. Variant II was chosen to test the ballistic properties of the combination of both structures. It was assumed that obtaining good ballistic properties will allow to eliminate the non-woven fabric and allow embroidering on the fabric, or it will be possible to make structures embroidered on the para-aramid fabric, which will allow to eliminate the non-woven substrate, which does not have any effect in dissipating the kinetic energy of the bullet and only unnecessarily increases the mass of the package ballistic.

**Figure 3:**
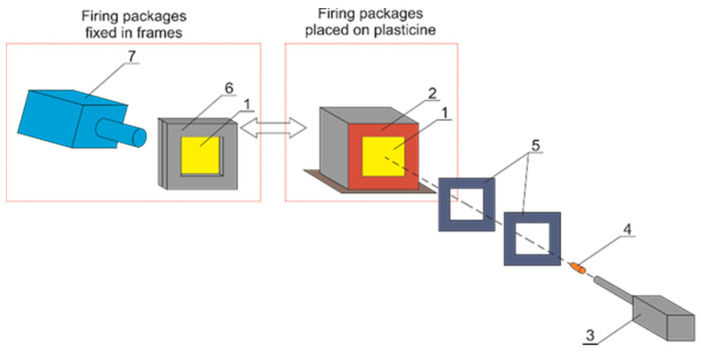
Diagram of the ballistic test stand (1—ballistic package; 2—mold with ballistic plasticine; 3—ballistic cannon; 4—bullet trajectory; 5—set of gates for measuring the velocity of the bullet; 6—steel clamping frames; 7—camera for quick registration).

Three packages of each variant were prepared for firing at the Ballistic Research Laboratory. The package was placed on the Roma No. 1 plasticine and fastened with velcro straps. In the first stage of the research, the transverse deformation of the packages was analyzed. At zero impact angle, one shot was fired with a commercially available 9 × 19 mm Parabellum bullet (Sellier & Bellot, Vlasim, Czech Republic) at a speed of 380 ± 3 m/s in the center of each package placed on a standardized Roma No. 1 plasticine substrate (Figure 3). The Parabellum 9 × 19 mm bullet was selected so that its fired speed fulfilled the minimum speed of bullet required during the testing of ballistic packages for NIJ level II according to NIJ standard 2008. The number of layers of the packages was selected based on literature reports, which show that the package made of pure 24 layers of Twaron CT709 fabrics, fired with a 9 × 19 mm Parabellum bullet at a speed of 364.85 m/s, exhibited a maximum deformation of the normalized plasticine substrate at the level of 30.5 mm [47]. Due to the higher velocity of the bullet used in the tests, the number of layers was increased to 26 so as to keep the deformation of the plasticine substrate at a similar level.

During the firing, the velocity of impact of the Parabellum 9 × 19 mm FMJ bullet was recorded through a set of gates to measure the velocity of the bullet. In order to measure the deformation of the ballistic substrate after firing, it was scanned with a laser distance sensor, the position of which in the XY axes was controlled by stepper motors. Each time an area measuring 18 cm × 18 cm was scanned, the center point was always positioned at the largest depression of the plasticine substrate. After firing, the ballistic packages were also analyzed for the number of shot layers. On this basis, the perforation coefficient was calculated as the ratio of the number of layers pierced to the number of all layers in the ballistic package (1), and the bullet expansion calculated as the ratio of the increase in the diameter of the bullet after hitting the ballistic package to the initial diameter of the bullet (2). The bullet diameters after impact were measured with calipers in six different places, and the average value was determined on the basis of the obtained measurements.

(1) $$\mathrm{Perforation}\;\mathrm{ratio}=\frac{\sum_{i=1}^{n}AD_{i}}{AD_{T}}\;$$

where $AD_{i}$ is the surface density of the *i*th perforated layer, *n* is the number of perforated layers, and $AD_{T}$ is the total surface density of the packet.

(2) $$\mathrm{Bullet}\;\mathrm{expansion}\;\left(\%\right)=\frac{D_{d}-D_{i}}{D_{i}}*100$$

where $D_{d}$ is the diameter of the deformed bullet after impact, and $D_{i}$ is the initial diameter of the bullet, i.e., 9 mm.

In the second stage of the research, the mechanisms of layer destruction during shelling were analyzed in detail. For this purpose, single woven and embroidered layers were attached to two steel frames and a single shot of a 9 × 19 mm Parabellum bullet was fired at a speed of 380 ± 3 m/s at the very center of the sample. The individual phases of the penetration of the layers by the bullet were recorded with a Cordin 550 high speed recording camera (Cordin Company, Salt Lake City, UT, USA) at a speed of 250.000 frames/s.

## 3. Results

**Figure 4:**
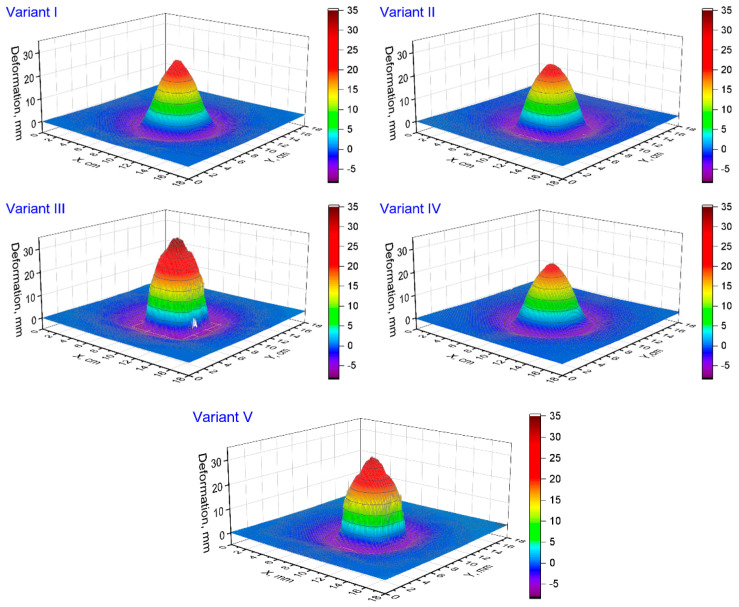
Deformation of the ballistic ground for individual variants of ballistic packages.

Figure 4 shows the deformation of the ballistic base after firing various variants of ballistic packages with a 9 × 19 mm Parabellum bullet at a speed of 380 ± 3 m/s. Comparing the deformation of the substrate during the firing of a package consisting of 26 layers of embroidered structures (Variant I) with the deformation during the firing of a package consisting of 26 layers of fabrics (Variant V), differences are visible both in the values of the maximum deformation and the shape of the indentation. In the case of the maximum deformation for Variant I and Variant V, it was respectively 25.9 and 30.5 mm. When analyzing the deformation shape, it should be stated that for Variant I, it has a spherical shape with a relatively sharp tip, and for Variant V, the shape of the deformation is similar to a pyramid. Such differences between the variants should be explained by the different structure of the structure of the layers because both structures have the same para-aramid threads, and the surface weight is comparable, not including the structure of the embroidered base material in the form of PP non-woven fabric and the weight of the threads embroidering the para-aramid yarn. The embroidered structure (Variant I) was made as two sets of threads in the configuration 0/90°. In this structure, there is no interlacing effect, so on the one hand, during the impact of the bullet, the threads immediately transfer high stresses, unlike the fabric (Variant V), where after the impact of the bullet, the threads first straighten and only then are able to transmit high stresses. The process of unfolding the threads in the fabric is accompanied by increased transverse deformation, which results in greater deformation of the plasticine base. On the other hand, studies of the stress wave propagation velocity in fabrics are known [48,49,50], which indicate that they are lower than the stress wave propagation velocity in the individual threads that they are made of. This is mainly due to the interwoven structure of the fabric. As there are no interlacing in the embroidered structure, it can be roughly assumed that the speed of stress wave propagation in this structure is comparable to that for a single para-aramid thread and greater than in the fabric. The greater propagation of the stress wave in the embroidered structure has a positive effect on the increase in the area of the stress wave distribution in the plane of the layers around the point of impact of the bullet. The work of the layers over a larger area also favors less lateral deformation of the ballistic packet and, consequently, less deformation of the plasticine substrate.

**Figure 5:**
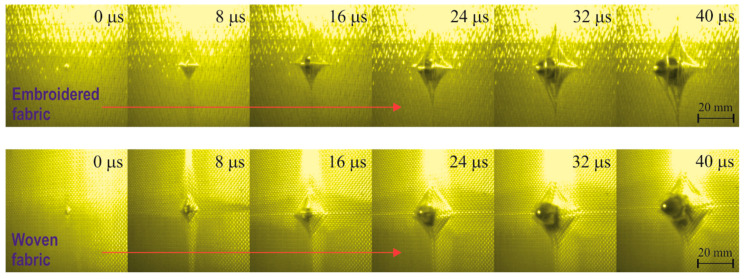
Stages of piercing a para-aramid embroidered and woven structure with a bullet, recorded with a high-speed camera.

The disadvantage of stacked structures compared to woven structures is the ease of spreading the threads during contact with the bullet face, which has already been noticed in previous studies [47]. The face of the bullet has a relatively small area, usually consisting of 2–3 warp and weft threads, and an ogive nose that promotes the separation of the threads. In the fabric, thread spreading in contact with the bullet is minimized due to jamming of threads in the interlacing. In the structures being laid, the threads are jammed, for example, with glue, but the holding force of these threads in contact with the bullet is low. For this reason, the obtained embroidered structures were analyzed in terms of thread spreading during the bullet impact. For this purpose, the bullet penetration of single woven and embroidered layers was imaged with a high-speed recording camera. The individual stages of penetrating the layers by the bullet are shown in Figure 5 at intervals of 8 µs.

**Figure 6:**
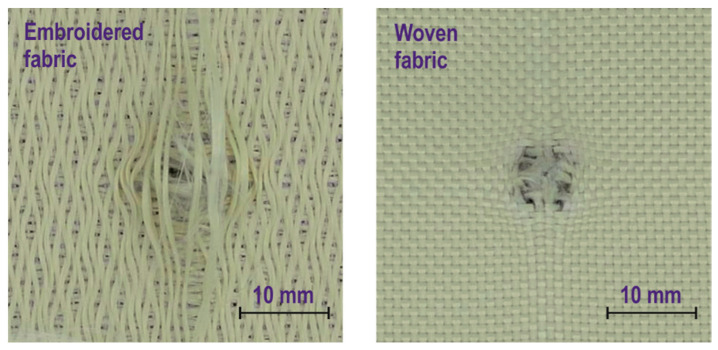
Photos of the embroidered and woven structure after the bullet was pierced.

As shown in Figure 5, in the case of piercing the woven structure 8 µs after the contact of the fabric with the bullet, the face of the bullet is visible, which is in contact with 2–3 threads pulled from the structure. These threads continue to break, and after 24 µs, the bullet resistance is only the friction of the side bullet shell against the sliding threads in the hole. In the case of piercing the embroidered structure, the face of the bullet is visible after 16 µs. It is clearly visible that the threads of the external system did not break but moved sideways along the conical part of the bullet. Only one thread contacts the forehead of the bullet, which begins to split in 24 µs and the bullet continues between its two parts without breaking them. It follows that from the external thread system of the embroidered structure, none of the threads broke and all the threads spread over the conical part of the bullet. Figure 6 shows pictures of embroidered and woven structures after the bullet was pierced. In the case of an embroidered structure, even the threads in contact with the face of the bullet did not break but spread apart. In the case of a woven structure, two and three broken threads appear vertically and horizontally, respectively. It follows that, as was noted in the case of structures containing glued strands of threads, also the embroidering of the threads does not prevent the threads from sliding apart during the impact of the bullet.

**Figure 7:**
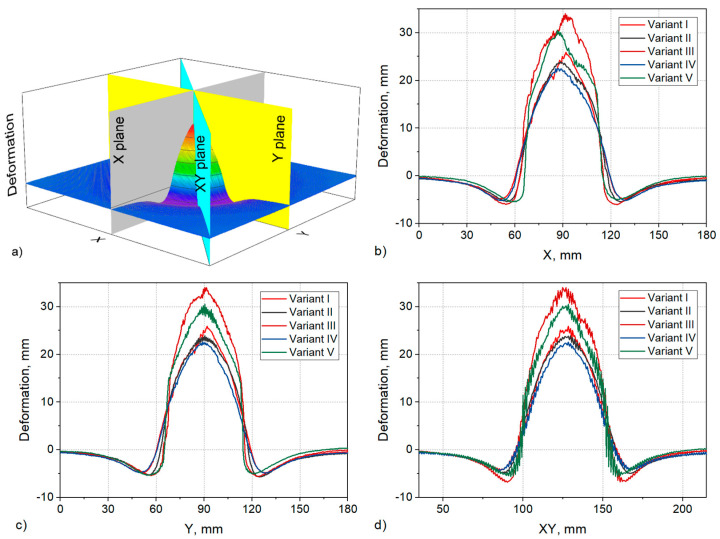
Cross sections of the plasticine substrate at the point of bullet impact for individual variants of ballistic packages: (**a**) diagram of the ballistic substrate division according to the planes in the X, Y and XY axes, (**b**) substrate cross sections in the X plane, (**c**) substrate cross sections in the Y plane, (**d**) substrate cross sections in the XY plane.

Embroidered structures, therefore, have two advantages and one disadvantage in terms of ballistic shock response. The advantages are straightened threads and the lack of interlacing, which is conducive to a high speed of propagation, which has a positive effect on a lower transverse deformation of the packet. The disadvantage of these structures is the sliding of the threads in contact with the front of the bullet, which adversely affects the number of shot layers in the ballistic package. It should be noted that the intensity of thread spreading depends on the angle of the conical surface pressing against the embroidered structure. In the case of a multilayer ballistic package, the spreading of threads in the subsequent layers will be smaller and it will not occur at all in the final layers. Woven structures have advantages and disadvantages in their response to ballistic impact, which are in contrast to the advantages and disadvantages of embroidered structures. The advantage here is the structure that is jammed by the weft and warp threads, which prevents the threads from sliding apart in contact with the front of the bullet. In turn, the disadvantages of this structure are the weft and warp threads and the lower speed of stress wave propagation due to the interwoven structure, which favors increased transverse deformation. Considering the advantages and disadvantages of both structures, it seems reasonable to create a multi-layer hybrid pack that will include structures woven on the front and embroidered on the back. Figure 4 shows the deformation of the plasticine base for the ballistic package (Variant IV) made in this way, which includes 13 layers woven on the front and 13 layers embroidered on the back. It is clearly visible that the maximum deformation depth is even smaller than for the package consisting of 26 embroidered layers (Variant I), and it was 22.6 mm. On the other hand, a very unfavorable variant, highlighting the disadvantages of woven and embroidered structures, would be the construction of a multi-layer hybrid package containing structures embroidered on the front and woven structures on the back. Figure 4 shows the deformation of the plasticine base for the package made in Variant III, with 13 embroidered layers on the front and 13 woven layers on the back. The maximum deformation of the substrate was even greater than in the case of firing a package consisting of only fabrics (Variant V) and amounted to 34.1 mm. The response of the hybrid packages made in Variants IV and III confirms the existence of advantages and disadvantages of embroidered and woven structures, but their proper management allows to significantly improve the ballistic properties of the packages. During the research, a hybrid package consisting of woven and embroidered structures arranged alternately (Variant II) was also subjected to fire. Figure 4 shows the deformation of the plasticine base for the package made in this variant. The maximum value of deformation was 23.9 mm and was 1.4 mm higher than in Variant IV, for which the lowest value of this deformation was obtained. The arrangement of alternating layers seems to be advantageous on the one hand because the package is stable when fired by a larger number of bullets; on the other hand, it is possible to make structures embroidered directly on the para-aramid fabric without the substrate. Then, the effect of increasing the mass of the package with the base of the embroidered structure, usually used in the form of PP nonwoven, will be eliminated. These studies, however, have not been implemented but are planned to follow. In an alternating layered bundle, embroidered structures arranged in front of the bundle are susceptible to thread separation upon contact with the bullet. Therefore, the dissipation of the bullet kinetic energy by these layers is limited. This has a significant influence on a slight increase in the maximum deformation of the ballistic base. The deformation of the ballistic base for individual variants of the ballistic packages was compared in cross sections. Figure 7 shows diagrams of cross sections passing through the point of bullet impact, made in the plane of both X and Y thread systems and in the 45° plane in relation to the thread systems (Figure 7a).

**Figure 8:**
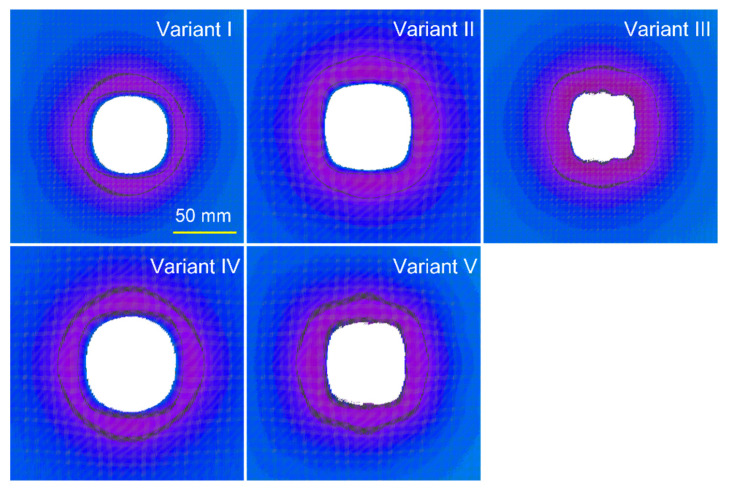
Inlet cavities at the zero level of the plasticine substrate when firing packages made in the assumed variants.

The individual cross sections presented in Figure 7 show, above all, the different, depending on the variant of the ballistic package, values of the maximum deformation of the plasticine substrate. The purpose of the analysis of these cross sections was to assess possible differences in the shape of deformation due to the asymmetry in the embroidered structure, which results from the overlapping of two thread systems, and in the X axis, the threads are always outside and in the Y axis, always inside. The situation is different in the CT709 fabric, where a plain weave is used, and there is full symmetry in the X and Y axes, assuming the same weft and warp threads are entered. When analyzing the width of the inlet chambers in the X and Y planes for the deformation value equal to zero (Figure 7b,c), no significant differences can be seen for the same ballistic package. The smallest dimensions of the inlet cavities and at the same time, the largest maximum deformations occur for Variants III and V. These are fabric-only ballistic packages (Variant V) and or with woven layers on the back (Variant III). The probable reason for this is the concentration of stresses in the fabric during the impact of the bullet on a smaller area in relation to the embroidered structures due to the lower speed of the stress wave propagation. The same effect is also visible in the XY plane. In turn, the widest inlet cavity is with the firing packages made in Variants II and IV. When firing these packages, the smallest maximum deformation of the ballistic substrate occurs simultaneously. It follows that due to the presence of a large number of embroidered structures in the rear part of the package, at the moment of the bullet impact, the stresses in these structures spread at a higher speed, which affects the distribution of the transverse deformation over a larger area of the ballistic package, at the same time, limiting the maximum values of this deformation. Figure 8 shows the shape of the inlet cavities at the zero level of the plasticine substrate when firing packages made in the assumed variants.

**Figure 9:**
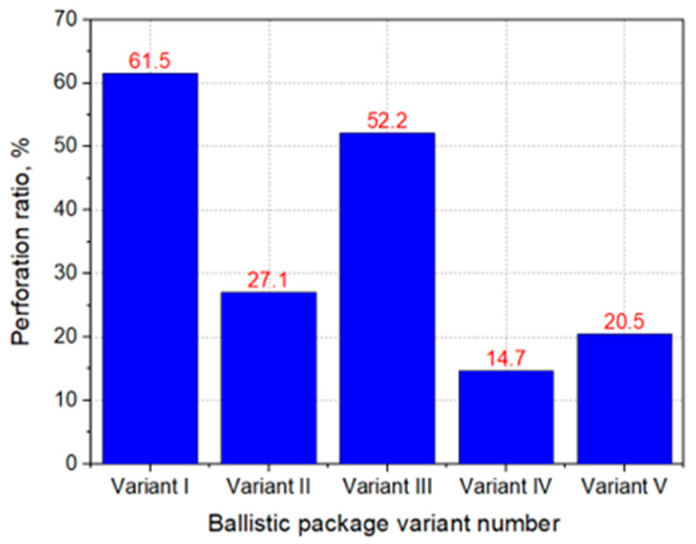
The perforation factor for individual variants of ballistic packages.

In the shape of these inlet cavities, the regularity is that embroidered structures contribute to circular and woven to square inlet cavities. The inlet cavity for Variant I (consisting of 26 embroidered layers) is practically round, while for Variant V (consisting of 26 woven layers), it is close to a square. Similarly, for Variant IV with 13 layers embroidered on the back, the cavity shape is round, and for Variant III with 13 layers woven on the back, the shape is close to a square. In the case of the cavity for Variant II, it has a wide, square–round shape, which is the result of the alternating arrangement of embroidered and woven layers. It seems that the cause of the larger area of deformation in the XY plane in relation to the X and Y planes for the fabrics placed in the back of the packet is the interwoven structure of the fabric, which contributes to the transfer of the transverse deformation to the thread areas not in direct contact with the face of the bullet. This is clearly visible in Figure 5, where in the case of a fabric, the base of the deformation cone has a shape similar to a square, and in the case of an embroidered structure, it has a shape similar to a curved four-point star. Figure 9 shows the degree of perforation for the tested variants of ballistic packages during their firing on a plasticine substrate.

**Figure 10:**
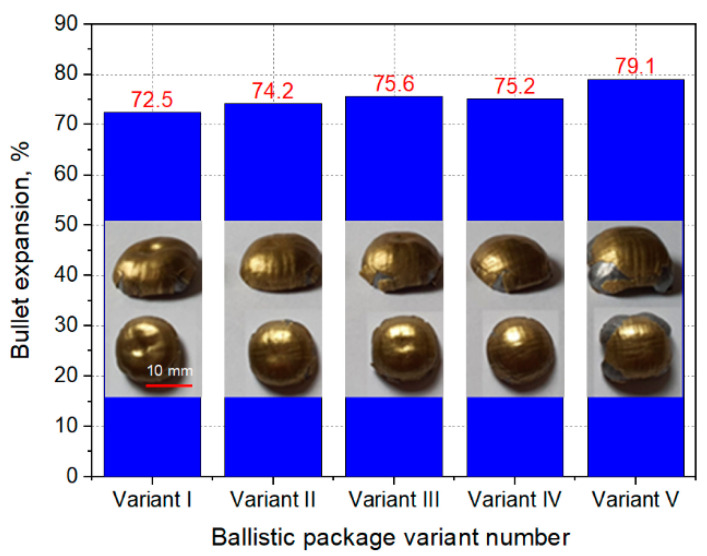
The expansion and view of the bullets after impact for individual variants of ballistic packages.

As shown in Figure 9, the highest degree of perforation occurs for the ballistic packages made in Variants I and III and amounts to 61.5 and 52.5%, respectively. First of all, this is due to the use of embroidered layers in the front part of the package from the side of the bullet impact. As shown earlier, these structures are characterized by the ability to spread the threads in contact with the bullet, and their ballistic effectiveness in such an arrangement is severely limited. In the case of the remaining packages, the perforation rates are much smaller, not exceeding 28%, which results from the fact that a large number of woven layers are placed in the front part of the package on the side of the bullet impact. Due to the interwoven structure, weft and warp threads do not move apart at the point of impact of the bullet, which means that even the initial layers significantly contribute to the increased ballistic effectiveness of the package. The lowest perforation rate of 14.7% occurs when firing a package made in Variant IV, for which there is also the smallest maximum deformation and the largest area of the inlet cavity. The bullet in this structure stops at the 5th layer, which should be considered an excellent result, as the bullet penetrates only 4 out of 26 layers of the packet. For comparison, for a package made only of fabrics (Variant V), the bullet stops at the 6–7th layer. Embroidered structures used in Variant IV quickly absorb the kinetic energy of the bullet due to the straightened threads, thus limiting the kinetic energy of the bullet affecting the woven structures. Figure 10 shows examples of bullets and the expansion of the bullets after hitting ballistic packets made in the assumed variants and placed on a plasticine base.

As can be seen in Figure 10, the bullet expansion after firing particular variants of ballistic packages is very similar and ranges from 72.5 to 79.1%. The obtained results of bullet expansion are so similar that it is difficult to ascribe to them the influence of the structure of layers and the arrangement of layers in individual ballistic packages.

Comparing the deformation of the ballistic plasticine after firing 26 layers of various ballistic packages with a 9 × 19 mm Parabellum bullet at a speed of 380 ± 3 m/s, the packages with Variants II and IV showed the lowest deformation values (23.9 mm for Variant II and 22.6 mm for Variant IV). Taking into account the ballistic tests with the Parabellum 9 × 19 mm bullet at the speed of 364.85 m/s of packets with 24 layers of Twaron CT709 fabric, the deformation of the normalized ballistic substrate was 30.5 mm [47]. Experimental and simulation tests on ballistic plasticine of packages made of 25 layers of Twaron CT709 fabric were performed by Kędzierski et al. The 9 × 19 mm Parabellum bullet with a speed of 371 m/s was used in the tests and simulations. In the experiment, the deformation of the ballistic plasticine was 33.6 mm, and in computer simulations the deformation was 35.6 mm [51]. The manufactured hybrid packages with embroidered structures showed better ballistic protection properties even at a higher velocity of the 9 × 19 mm Parabellum bullet, compared to the reported results of research on packages made of Twaron CT709 fabric.

## 4. Conclusions

1. The article presents ballistic studies of para-aramid embroidered structures, which so far have not been considered as layers of textile ballistic packages. Comparative ballistics studies of embroidered and woven structures have shown that they have opposing advantages and disadvantages in terms of ballistic shock response.
2. In the case of embroidered structures, the advantages are straight threads and the lack of interlacing, which is conducive to high propagation speed, which has a positive effect on the lower transverse deformation of the bundle. The disadvantage of these structures is the sliding of the threads in contact with the front of the bullet, which adversely affects the number of shot layers in the ballistic package.
3. In the case of woven structures, the advantage is the structure jammed by the interweaving of the weft and warp threads, which prevents the threads from sliding apart in contact with the face of the bullet, and the disadvantages of this structure are that the weft and warp threads are wrapped in, and the speed of stress wave propagation is lower due to the interwoven structure, which promotes increased transverse deformation.
4. Considering the advantages and disadvantages of both structures, it is advantageous to assemble a multilayer hybrid bundle with woven structures on the front and embroidered structures on the back, such as the tested variant of the IV package. This package achieved the smallest maximum plasticine deformation of 22.6 mm, the largest inlet cavity area and the smallest perforation rate of 14.7%. For comparison, for a ballistic package with a traditional design, composed only of woven structures, the values were 30.5 mm, respectively, the area of the inlet cavity was smaller, and the perforation rate was 20.5%.
5. Further research into embroidered structures for use in ballistic packages is warranted in three directions:
  - The execution of structures embroidered on para-aramid fabric, which will allow to eliminate the non-woven substrate, which does not have any effect in dissipating the kinetic energy of the bullet and only unnecessarily increases the weight of the ballistic package;
  - The execution of multi-axis embroidered structures;
  - Numerical modeling of a bullet impact in embroidered and hybrid structures and finding the most optimal arrangement of the woven and embroidered layers in a ballistic package.

## Data Availability

The data presented in this study are available in the databases of the authors at the Lodz University of Technology.
